# Shared Decision Making at the Limit of Viability: A Blueprint for Physician Action

**DOI:** 10.1371/journal.pone.0166151

**Published:** 2016-11-28

**Authors:** Thierry Daboval, Sarah Shidler, Daniel Thomas

**Affiliations:** 1 Children’s hospital of Eastern Ontario. Department Pediatrics. The Ottawa Hospital, Department of Obstetrics and Gynecology. University of Ottawa, Ottawa, Canada; 2 Department of Health Science Sciences, University of Quebec in Abitibi-Temiscamingue, Rouyn-Noranda, Québec, Canada; 3 Department of Human Sciences, University of Quebec in Abitibi-Temiscamingue, Rouyn-Noranda, Québec, Canada; University of British Columbia, CANADA

## Abstract

**Objective:**

To document interactions during the antenatal consultation between parents and neonatologist that parents linked to their satisfaction with their participation in shared decision making for their infant at risk of being born at the limit of viability.

**Methods:**

This multiple-case ethnomethodological qualitative research study, included mothers admitted for a threatened premature delivery between 20^0/7^ and 26^6/7^ weeks gestation, the father, and the staff neonatologist conducting the clinical antenatal consultation. Content analysis of an audiotaped post-antenatal consultation interview with parents obtained their satisfaction scores as well as their comments on physician actions that facilitated their desired participation.

**Results:**

Five cases, each called a “system—infant at risk”, included 10 parents and 6 neonatologists. From the interviews emerged a blueprint for action by physicians, including communication strategies that parents say facilitated their participation in decision making; such as building trustworthy physician-parent relationships, providing "balanced" information, offering choices, and allowing time to think.

**Conclusion:**

Parent descriptions indicate that the opportunity to participate to their satisfaction in the clinical antenatal consultation depends on how the physician interacts with them.

**Practice implications:**

The parent-identified communication strategies facilitate shared decision making regarding treatment in the best interest of the infant at risk to be born at the limit of viability.

## Introduction

Shared decision making, now included in family-centered care and promoted by major professional societies [1–4], implies a discussion between parents and physicians regarding their respective criteria for defining the “best interest” of the infant at risk of being born at the limit of viability. Research shows that each participant in this decision-making process may use different criteria. Parents are guided by hope, spiritual values [5], their interpretation of the information available, their desired involvement in decision making, the support received and the trust built with the neonatologist [6]. Most neonatologists consider that the determination of the best interest of the infant should be primarily guided by the best medical evidence regarding the probabilities of survival and morbidity [7], what some authors call the “best evidence model of shared decision making” [8]. However, this model has not been studied in clinical situations where a vulnerable infant is potentially facing **life** with a range of resultant qualities of that life (light to heavy handicaps) or **death** (at the end of palliative/comfort care) [9].

In order to ethically justify the final decision regarding treatments in the best interest of such infants, these crucial and very sensitive discussions should follow certain "rules" to ensure open and honest participation of each actor [10]. Thus, shared decision making for severely compromised neonates must exceed in scope the mere discussion of medical best evidence on risks and benefits concerning treatment choices and must also include parental values regarding such matters as religion, spirituality, family, and hope not necessarily linked to medical evidence [5].

Parents want to participate in the decision-making process [11–16] and some parents feel frustrated or have regrets when their involvement in the decision-making process is less than they had desired [12,17–20]. Each parent’s desire to participate is different and studies show that the neonatologist cannot accurately judge in advance the extent to which parents wish to participate [21].

There is a recognized need for clinical studies examining the characteristics of communication that facilitate the participation that parents desire in the decision-making process [4]. The object of the present study was to describe the characteristics of the communication that parents identify as facilitating their desired participation in decision making during the antenatal consultations between a neonatologist, pregnant mother and father–where these “life or death” decisions concerning their infant at risk of being born at the limit of viability are discussed.

These results come from a larger observational study of physician-parent communication during antenatal consultations [22]. Presented here are parents’ descriptions, obtained during a post-antenatal consultation (PAC) interview shortly after the clinical antenatal consultation, of physician actions that facilitated, or not, their participation in decision making.

## Methods

### Research Strategy

Seeking to discover how parents’ describe, soon after the antenatal consultation, those aspects that facilitated, or not, their desired participation in decision making, a prospective, ethnomethodological multiple-case study strategy was used. This research is ethnomethodological in that it studies aspects of how individuals communicate, reason, and make decisions in the moment in a specific cultural context [23,24] (here, the antenatal consultation during the hospitalization of a woman at risk of delivering a baby at the limit of viability). Due to time restraints and the limited number of parents’ meeting the inclusion criteria, a multiple-case study design with semi-directed interviews was chosen to allow an in-depth exploration of the interactions during a number of different antenatal consultations [25]. In the post antenatal consultation interviews, rather than theoretical saturation of themes, rich descriptions of parental points of view regarding satisfactory participation in a shared decision making process were sought.

### Recruitment Criteria

Participants were recruited in the department of Obstetrics and Gynecology in a tertiary care teaching center in Ottawa (Canada) over a period of 6 months. Included were the principal decision makers in the context of a clinical antenatal consultation: the mother referred by her obstetrician and admitted for a threatened premature delivery between 20^0/7^ and 26^6/7^ weeks gestation, the father, and the staff neonatologist or senior fellow in neonatal perinatal medicine (NPM). We defined this observational unit (mother, father and neonatologist or fellow in NPM) as a “system–infant at risk” (hereafter called system). Excluded were parents less than 18 years old, medically unstable mothers, parents where one of the two refused to participate, mothers expected to deliver within the next 6 hours after the antenatal consultation, parents not speaking fluently either French or English, same-sex parents, and pregnant mothers with known malformed fetus.

### Ethical Considerations

Parents meeting the recruitment criteria were approached by the staff neonatologist or senior fellow (NPM) to participate in the order they were referred by the obstetrician for clinical antenatal consultation due to the risk of a premature birth. The study was explained to the parents, distinguishing the normal clinical aspects and the research-related PAC interview. The parents were assured that their clinical treatment would not be affected or delayed due to their consent or refusal to participate. The parents were given time to think about and discuss together their wish to participate. After the parents agreed to participate, the neonatologist or the fellow in NPM was contacted for consent. The principal investigator who conducted the PAC interviews is a neonatologist working full-time in the neonatal intensive care unit. During this study, to avoid confusion, bias and conflict of interest he was not involved in the antenatal consultations studied. Parents were not recruited if the principal investigator was on clinical duty in the neonatal intensive care unit. Recruited parents may have been involved with the principal investigator as part of the team caring for their infant born after their participation in the study.

Ethics approval was obtained from the Research Ethics Boards of both the Ottawa Hospital Research Institute and the Université du Québec en Abitibi-Témiscamingue (as this study was part of the principal investigator’s Master’s Degree). All participants including parents and neonatologists provided their written informed consent to participate before any further research procedures.

### Data Collection

The larger study included two specific phases: First, videotaping the regular clinical antenatal consultation (results not shown here are described in reference [22]) and secondly, audio taping the research-specific PAC interview. Both interviews were transcribed and verified for authenticity by the transcriber and the principal investigator.

The *clinical antenatal consultation* between parents and a staff neonatologist and/or an NPM fellow followed, but not strictly, an antenatal consultation format (ACF) [26,27] developed by three teaching neonatologists responsible for integrated clinical ethics training, based on the principles of shared decision making [1], discourse ethics [28], communication theory [29,30] and a review of the literature [6,31–35]. The ACF allows for two separate encounters with at least one done by a staff neonatologist unless unexpected circumstances happen such as the baby is born before or the mother has been discharged. The ACF format outlines general topics and interview procedure, but not the actual communication strategies of the clinicians. The content of these communication strategies and their impact on parents’ satisfaction with their participation were solicited in the PAC interviews.

The *PAC interviews*, consisting of a 20 to 66 minute in-depth, semi-directive, face-to-face interview of both parents together by the principal investigator, were conducted either in French or English, according to the parents’ preference, within 4 hours following the end of the final part of the antenatal consultation done by the staff neonatologist/fellow in NPM. For system # 5, a staff neonatologist did not have the time to meet with the parents before the mother was discharged from hospital.

After weighing disadvantages and benefits, the investigators agreed to proceed with interviewing parents together instead of individual interviews with the benefit of each parent adding more information to the partner’s description of satisfaction about participation in the decision making process. The PAC interview followed an interview guide that focused on parent satisfaction concerning their participation in the decision-making process and on the actions of the physicians that parents said influenced their degree of satisfaction. Satisfaction was measured with a 0 to 10 standard visual analog scale (VAS) [36] by each parent scoring their own form silently and separately; 0 being “totally dissatisfied with my participation” and 10 being “totally satisfied with my participation.” The score guided the interview of the parents: if the score was equal to or less than 7, the interviewer asked “what should have been done to increase the score by 1 or 2 points?”, and if the score was 8 or more the question was: “What elements during the consultation explain your score?”. We also asked each neonatologist and fellow in NPM who did the clinical antenatal consultation to evaluate their own perception of parental satisfaction concerning participation in the decision-making process using a 0 to 10 VAS similar to the one used with parents.

Written data collection forms were also used to facilitate collating information such as the date of time of both part on the antenatal consultation, details on who conducted the antenatal consultation, gestational age of the pregnancy of the mother, the age of both parents, the date and time (start and end) of the post antenatal consultation and if referral to the social worker service was made as result of the participation to the research.

### Data Analysis

Content and iterative analyses from a constructivist orientation were applied to the transcripts with the goal of obtaining a rich description of the parents’ perspective of the antenatal consultation. Theoretical saturation was not possible due to the small number of parents included. The initial paraphrastic codes generated from the words used by the parents in the PAC interview led to the identification of common themes explaining their satisfaction with their participation in the decision-making process. A constant comparative method was used to group these themes and identify conceptual categories that described parent satisfaction between each system. An on-going reflexive process started as the data were being collected. After the first interview it was difficult to ascertain whether parents were evaluating on the VAS their *satisfaction with their participation* in the decision-making process or their satisfaction with the decision. This observation led to the modification of the interview guide by asking first questions about what was discussed during the antenatal consultation and the way they participated in the decision and then, in the middle of the interview presenting the VAS. This change was made to ensure that parents were evaluating their satisfaction with their participation. Moreover, in keeping with the constant comparative method of data collection and analysis, some of the emerging themes that were identified through the previous interviews guided subsequent interviews. For example, at the beginning of the coding process, evidence of gender differences was sought. However, it soon became apparent that parents were agreeing among themselves and were sharing the same viewpoint on the determinants of their satisfaction.

The principal investigator coded all the transcripts. Being an experienced neonatologist, the principal investigator had the advantage of a theoretical sensitivity to the various factors that could influence parents’ participation in the decision-making process, but he also had the disadvantage of his own preconceptions that could bias the analysis of the data. As counter-coding strategy to ensure the validity of the results, the principal investigator met twice for a total of 6 hours to review the coding and related transcripts and to discuss potential biases with his two co-authors who came from very different backgrounds: one in law/clinical psychology/bioethics and the other in sociology. Modifications were made as needed until agreement was reached. To aid the coding and organization of the data, NVivo qualitative data analysis software (QSR International Pty Ltd © Version 9.0, 2011) was used.

## Results and Interpretation

### Participants

From December 2011 to May 2012, twelve mothers at risk to deliver at the limit of viability meeting the inclusion criteria were referred by their obstetrician. Half of them and their partner provided their written informed consent to participate; however, one of these mothers delivered before the clinical antenatal consultation. All neonatologists and fellows involved in the antenatal consultation for these parents also provided their written informed consent prior to the research procedures started. In total, 5 systems consisting of 16 participants (5 mothers, 5 fathers and 6 neonatologists or fellows in NPM) were included in the study. Participating parents varied in age (ranging from 20 to 37 years) and gestational age (from 22^+6/7^ to 26^+0/7^ weeks). The demographic characteristics of the participants appear in Table 1.

**Table 1:** Description of the participants, parents’ participation satisfaction scores and number of antenatal consultation.

System	Gestational age (weeks)	Participants	Age (years)	Parents satisfaction (—/10)
#2	22+6/7 (Twin gestation)	Father	22	9
		Mother	20	9
		Fellow in NPM**	-	8
		MD*	-	8
#4	24+1/7	Father	37	10
		Mother	30	9
		MD*	-	9
#1	24+4/7	Father	25	10
		Mother	24	10
		MD*	-	9
#6	25+3/7	Father	33	9
		Mother	30	8
		MD*	-	10
#5	26+0/7	Father	25	2
		Mother	26	3
		Fellow in NPM**	-	7

* MD: Physician (MD) neonatologist

** NPM: Neonatal Perinatal Medicine

### Parents’ perception of their role in the decision-making process

The variability and the complexity of the parents’ perception of the role of each participant in the decision-making process was noticeable. The analysis of the verbatim of the PAC interviews demonstrated that parents wished to participate in the decision-making process since they felt directly concerned, albeit in distinctive ways, vis-à-vis their partner and vis-à-vis the physician for each system.

Vis-à-vis the physician; the parents wanted, to varying degrees, the opinion or guidance of the health care professional from "it is a parent decision" to "the physician making the decision, while maintaining a desire to understand the basis of that opinion". The different perspectives presented:

It is a parent decision notwithstanding physician’s opinion:

*« I understand that*, *but it is still my decision because it is my child*, *right?*…,… *I appreciate a doctor to say they do not think it will work*, *but you can still try*. *I am glad they have an opinion*, *but I have one too*. *»* (Mother—System #2)

It is a parent decision based on selected information:

*« If the baby was coming*, *I would give a chance to my child*, *I said it with my words*. *[*…*] The doctors know what is good for the baby*. *So we share the information and I triage them*. *I am taking what is good for my baby and me*. *»* (Mother—System #4)

It is parent decision but physician may have an opportunity to make a different choice:

*« I’m glad that we do have a [a part in the] decision*, *because when it comes down to it*, *it is our baby*, *but also the fact [*…*] that we get the professional side of it as well*. (Father—System #1) *[*…*] Even if we say yes*, *go for it*, *they won’t just do it and that’s all*, *they are going to take into consideration the baby’s health also*. (Mother—System #1)

It is a physician decision but want to understand the rationale:

*« The healthcare practitioners’ advice would weigh very strongly on our decision because we do not know this stuff*. *[*…*] You know*, *we basically were going to leave it in the medical professionals’ hands to tell us what to do and we would do it*. *[*…*] I want to know what they are doing*, *but I want to understand why they are doing it*. *»* (Mother—System #6)

The parents who were not satisfied with their participation felt that the physician had all the control over treatment decisions.

*« I did not perceive that I had control of this*, *because I am not the physician*, *I am not the one performing the resuscitation*, *I feel that I don't have control of this*. *…*. *»* (Mother—System #5)

If the physician had explored their wishes to participate (as was done in the PAC interview), he would have discovered that these parents felt that the decision should have been theirs and they should have been told that. They felt they did not have the opportunity to participate as they would have wished.

*« If the decision belongs to the parents*, *yes*, *in this case*, *yes [*…*]*. *It would have been nice to have been told that ‘in this case there are these risks and the decision is yours if you want to keep going or not’*… *»* (Mother—System #5)

Vis-à-vis their partner; some parents–both mothers and fathers–felt that the mother has a more predominant role in the decision:

*« Honestly*, *I feel like I have more authority over my children then he (her partner) does because I feel like they (twins) are mine*. » (Mother–System #2)

*« She is carrying the baby*, *that is why I say all decision she will make […] her decision will be mine*. *»* (Father–System #4)

For others the responsibility of the decision is shared equally between the partners.

*« I don’t want to influence you*, *but I think that is fifty -fifty*. » (Mother) […] *« I am pretty sure that we are on the same page on this*. » (Father—System #5)

*« […] it’s not just one person*, *it’s both of us*. *It’s both our baby*, *so we have to discuss what…it’s not just one person that will make the final decision*. *»* (Father—System #1)

Notwithstanding the complexity and the variability on how each parent wished to participate in the decision-making process to the point that each system was distinctive, parents described similar physician actions relating to their satisfaction with their participation in the decision-making process.

### Parent satisfaction with their participation

The parents in 4 systems (# 1, 2, 4 and 6) scored their satisfaction with their participation in the decision-making process between 8 and 10 on the VAS. In one system (# 5), the father and the mother scores were 2 and 3 respectively, which we characterize as “dissatisfied” because of the great difference from the other systems and considering their PAC verbatim. Surprisingly, even though the parents rated the scores individually and silently; scores were similar between the two partners for all 5 systems. All physicians thought that parents were satisfied, giving perceived parent satisfaction scores between 7 and 10 including for the parents (system # 5) who were dissatisfied. The participation satisfaction scores for all participants of each of the 5 systems can be found in Table 1.

### Physician actions relating to parent satisfaction with their participation

Themes identified from the analysis of the verbatim transcription of the PAC interviews relating to physician actions that facilitated parent satisfaction with their participation fall into four broad categories: 1. Building a trustworthy parent–physician relationship, 2. Providing balanced information, 3. Offering choices regarding treatments, 4. Allowing time to think. Categories are presented in Table 2 in order of importance based how often they were identified in these 5 systems; “building a trustworthy parent–physician relationship” was clearly described in all 5 systems and “allowing time to think” in only 2 systems. However, this “order of importance” may be unique to these 5 systems. Quotes from parents obtained during the PAC interviews identifying the physician actions and the communication strategies described below are presented in Table 2.

**Table 2:** Physician actions and communication strategies identified by parents as facilitating their satisfaction with their participation in decision making.

Actions	Communication strategies to facilitate parent participation	Quotes from parent interviews
**1. Building a trustworthy parent–physician relationship**	Demonstrate empathy by validating parents’ emotional reactions and thoughts, when appropriate use gentle touch, show quietness, peace.	*« His demeanor is good and he cares about the person*, *I can tell*. *He is very into the conversation and he wants to help people*. *» […] « You need to show people that they are dealing with hard situations and you need to be sensitive to how they are going to react*. *» (System #2)*
		*[…] « Maybe just walk in with a softer voice*. *» (System #2)*
		*« She is very empathetic*, *so you have a connection of trust with her*. *» [*.* *.* *.*] « I also loved the quietness when she (the doctor) talked to us*. *It was simple*, *she was*, *I don't know*.* *.* *. *in peace*.*» (System #4)*
		*« The doctor treated me with a lot of empathy … she did not hesitate to touch me*. *»(System #5)*
		*« Yes*, *it was more of a deep dive conversation with*, *you know*, *more sit down and a little more intimate conversation with the doctor » (System #6)*
	Support parents—Ask parents how they want to be supported	*« It was comfortable*, *she interacted with us and she made us feel that she was on our side*. *It wasn’t that she was just kind of ignoring us*, *she was right there with the both of us*. *» (System #1)*
		*« He is supportive and he asked me that too*. *He asked me how I thought he could support me and I said to basically tell me the truth and just be there for me*. *» (System #2)*
		*« When we declared our choice*, *she said that she would have done the same*. *… She did not pre-empt us*. *» (System #4)*
	Make visual contact	*« She kept*, *you know*, *eye contact with both of us*. *She didn’t just look at me or just at him*, *and she went back and forth » (System #1)*
		*« At the same time they cannot tell if you are upset*, *not looking at me*. *» (System #2)*
		*« Yes*, *the eyes …*, *the eye contact was authentic […] Like the eyes were saying*: *listen*, *that’s just it*. *» (System #4)*
	Demonstrate competence—Review and remember major details from the mother's medical chart.	*« You can see that she is very knowledgeable*, *she knows what she is doing*. *» (System #4)*
		« *I like the fact that she made a little bit of research*, *like had notes on her ultrasound that she knew a bit what was going on*, *so at least then we weren’t totally in the dark*. *» (System #1)*
		*« She seemed to know about me*. *[*.* *.* *.*] She had read my chart and that meant a lot to me because she made the effort to understand my case*. *» (System #6)*
	Listen carefully in silence—Pay attention to parents concerns	*« She listened*. *You know*, *she—I would interrupt her to ask a question and she did not show any annoyance like ‘there he goes again asking me another question’ sort of demeanor*. *… She respectfully answered any question be it stupid or not*, *you know*. *» (System #6)*
	Pace the conversation to parents’ indications or needs	*« He kept reassuring us that we are not just going to stay negative here*. *He said that if we wanted we could stop*. *[*.* *.* *.*] He just stopped and he went on to the next thing*, *you know? I asked him to stop and he stopped and went on to the next thing*. *» (System #2)*
	Alternate the “expert” stance	*« He kept throwing the conversation back at me*, *but in a better way*. *I kept going back into the conversation*. *» (System #2)*
**2. Providing Balanced Information**	Describe risks and advantages, positive and negative outcomes of the situation	*« You have this chance or you might not even have it that bad*, *it’s just reassuring that we can see both aspects*, *bad and good*. *» (System #1)*
		*« If I could know that they will talk to me and guide me and tell me the pros and cons and these are the major things I will be looking at—mostly cons if you make a decision*. *That would have made me feel better about being involved in the decision-making process*. *» (System #6)*
	Describe the statistical information and the limits of their applicability	*« Knowing the numbers gives me something to work with*. *It is not vague*, *it is very clear to me and numbers are numbers and stand for something*, *right? It helps us to cope and is a very useful coping mechanism for us*. *» (System #6)*
		*« I am sure everyone around the world knows that stats are just stats*, *you know? […] Do you want to believe in the stats 100 percent or do you want to—I guarantee you the stats from 2010–11 are a way different than 2000*. *They are completely different because stats change all of the time*. *» (System #2*)
	Provide clear, direct and truthful information even though devastating	*« [*.* *.* *.*] It is not something you want to hear*, *it is not something anyone wants to hear that your children might not make it*. *That is not cool*, *you know*, *but I am glad that he told me because now I know*. *[*.* *.* *.*] I appreciate doctors who tell me the truth*. *I do not want people to hold back information even if it makes me cry*. *» (System #2)*
	Describe what will happen to their baby,–step-by-step	*« This is what we were going to go through and just more or less a high level*, *so the baby will get wrapped in plastic or some form of plastic and taken directly into NICU and I am able to go with in case I need to make any decisions*. *They would take blood almost on a daily basis*, *which would probably mean they would need a blood transfusion and things like that*. *You know*, *little things more of a high level overview of the step-by-step sorts of things of what would happen*. *» (System #6)*
	Allow and answer parents’ questions	*« She answered all the questions that we did ask*, *even though there were only a few*, *but she went above and beyond and spoke about even*, *even out of the weeks I’m in right now*. *» (System #1)*
**3. Offering choices**	Explain options that parents could choose from	*« You (the physician) made me understand my options and I weighed my options » (System #2)*
		*« It wasn’t like a “oh well*, *you have to take this”…*, *we had choices*. *They would look at all options for the baby and me*, *for all of us*, *because if anything would happen to the baby*, *how we would feel afterwards…*. *she gave us options that we could choose from*. *» (System #1)*
	Discuss treatment options related to parent concerns and values (to prevent parent disengagement)	*« [*.* *.* *.*]*, *if the babies do not make it we would suggest just wrapping them up and cuddle them’*. *That is why I cried; I took offence at his saying it just like that—just wrap up my kid*. *… It was a shock*. *[…] just knowing that our kids are not going to make it—most parents would not even want to hear the rest of it*. *» (Father—System #2) «That is it; he (the father) shut his ears off after*. *[…] I know*, *as soon as he (the physician) said that*, *his (the father) head turned and it was like oh-oh » (Mother—System#2) «[…]*.* *.* *. *maybe put the three options in a different format instead of going straight to the negatives*.*» (Father—System #2)*
	Consider discussing options even though different from the parents' opinion	*« I appreciate a doctor to say they do not think it will work*, *but you can still try*. *I am glad they have an opinion*, *but I have one too*. *» (System #2)*
		*« But in same time*, *the doctors know what is good for the baby*. *So we share the information and I triage them*. *» (System #4)*
	Consider discussing other options even though not described in guidelines	*«It would have been nice to have been told that in this case there are these risks and "the decision is yours" if you want to keep going or not*.* *.* *.* » [*.* *.* *.*] « To tackle this in the discussion that we had with the other doctor*, *she never told us about this … that is possible that we have to make choices… if “baby’s name” is born premature*. *She never said that [*.* *.* *.*] Because*, *want it or not*, *there are no choices*, *it’s like*, *she (the mother) will start to bleed and her life will be jeopardized*, *we don’t have the choice to do it*. *We will do it*, *and then*, *that is life … (emotion) … sorry… » (System #5)*
**4. Allowing time to think**	Offer time for the parents to discuss among themselves alone about the information and treatment options—No need for an immediate answer	*« That we didn’t have to decide right away*, *but it was just like “here are your options*, *think about it” and that’s how…it wasn’t just pushing us in*. *» (System #1)*
	Conduct a follow-up visit	*« It is a lot; I would not have remembered everything she had said today and would never have asked those questions if she had come in last night*. *I would have been too tired and scared*.*[*.* *.* *.*] Then*, *being able to sit down with her today it felt like we know what they said and it also gave us that opportunity to think about questions that we had or to ask for clarification on anything I had not thought of before*. *» (System #6)*

#### 1. Building trustworthy parent–physician relationship

The development of trust between parents and the physician was mentioned multiple times by parents in PAC interviews of all 5 systems to explain their satisfaction of their participation in the decision-making process. As laid out in Table 2, parents gave many different examples to illustrate the importance of the establishment of a true relationship with the neonatologist. It was noticeable that each parent described specific physician actions to explain the emergence of a "true connection" with the neonatologist. Being able to "*to try and put themselves* [physicians] *in the situation that they are walking into*" (System #2) or being sensitive to parents' situation to be supportive or empathetic seemed important. Many different non-verbal actions were often mentioned including the quality of the visual contact, being able to sit, being personable, being calm in the tone of the voice and gestures, and listening to parents' concerns. The ability to demonstrate competence not only by being knowledgeable but also showing an understanding of the mother’s specific medical history was noted. Also mentioned was keeping parents in the conversation by asking them how they would like to be supported, by facilitating questions from them, and by avoiding interruptions or long explanations; as System #5 described it as “*It was like a long fax*”.

#### 2. Providing balanced information

Hearing clear, direct, and truthful information from the physician even though it could be devastating was important to parents. Parents found it reassuring that the physician was able to keep a balance between the negative and the positive potential outcomes. More than the statistics describing the best evidence and the limits of their applicability, most parents wanted information regarding what would happen, step-by-step, to their baby after birth and to be assured that they will be kept posted on how their baby reacts to the care provided. It was also important to parents to be allowed to ask questions and to receive answers as well as to have access to tools to help them remember the information received.

#### 3. Offering choices regarding treatment options

Explaining treatment options including palliative care, limitation of resuscitation and intensive care, aligned to the best evidence of risks and benefits for their infant in a way that parents could understand and also allowing parents to choose options influenced their satisfaction concerning their participation in the decision-making process.

When parents felt their concerns and values were not respected, they disengaged, which jeopardized their opportunity to participate. This could be due to the content or the manner of presentation of possible treatment options. Respectfully presented, the physician’s expert opinion about a specific option could be welcome even though it was different from the parents’ opinion. When alternatives were not discussed, parents felt they had no choices and were dissatisfied.

#### 4. Allowing time to think

Parents appreciated when the physician allowed them time to think about the options presented without rushing them to make a decision. Giving parents an opportunity to think about the information received and the available choices allowed them not only to digest the volume and the complexity of information but also to identify their questions that would clarify their understanding. That the antenatal consultation was conducted in two parts divided by a pause was mentioned as facilitating parents’ participation.

## Discussion

**Fig 1:**
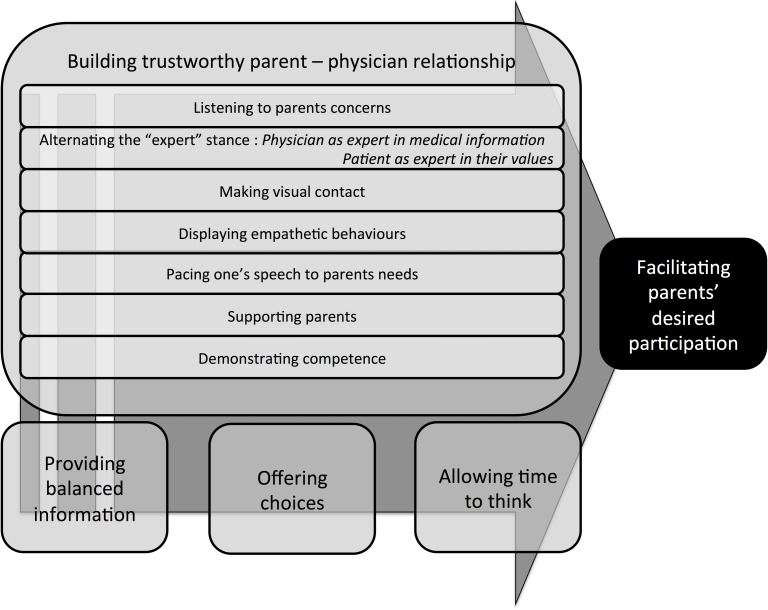
Blueprint for facilitating parents’ desired participation in shared decision making.

This study provides useful insights about physician actions identified by parents as facilitating, or not, their desired participation in antenatal consultations. Taken all together, the actions described by parents provide a blueprint (Fig 1) for physicians to facilitate parent participation to the extent that each parent desires. If appropriate physician communication strategies are lacking in the clinical consultation, parents may feel disappointed or judged to the point of disengaging, becoming silent, which could be interpreted as a lack of desire to participate when in fact they are reacting to the actions of the physician.

One of the most important observations made through this study was that even though there was a great complexity in how parents describe their role in the decision-making process, with only a few gender differences both fathers and mothers described comparable determinants of their satisfaction in their participation in the decision-making process.

Two of the systems were distinctive as a fellow was involved in the antenatal consultation. One in particular (system #5), parents dissatisfied with their participation in the decision making process met only the fellow. For this particular system (#5), two different aspects were noticed after reviewing the videotaping of the consultation (see reference [22] for more details): First, the frequency of interruptions made by the fellow was high compared to the other systems which illustrates an inability of the fellow to alternate the “expert” stance to the point that the parents disengaged from the conversation, and secondly, no choice other than full intensive care option was discussed with the parents. In the PAC it was clear that the parents would have wished to discuss an alternative option related to their concerns. These unique characteristics of this system (#5) reinforced our findings that other parents found their participation was facilitated by the clinician discussing choices or options with parents by alternating with them the “expert” stance, mentioning that it helped to build a trustworthy relationship between them. Second, also in this system (#5), the dissonance between the fellow’s perception of level of parents’ satisfaction of their participation and the actual satisfaction scored by both parents was remarkable. This finding is similar to other research in demonstrating discrepancies between parents and physicians regarding both how parents want to participate [21] and how shared information is recalled or interpreted [6]. However, that the interview of system #5 was conducted by a fellow does not diminish the importance of what we learn from this case. The object of this study was not to discover the “best” clinician, but rather to discover those clinician *actions* or *communication strategies* that parents say influence and facilitate their participation in decision making.

Our findings add further pertinent information to prior research concerning parent participation in a shared decision making process.

First, building a trustworthy parent-physician relationship seemed crucial for parents who are at risk to give birth to an extremely premature infant. Parents’ trust in their physician has been shown not only to influence decision-making process but also to allow parents to be reassured and to better cope with the uncertainty associated with the difficult decisions to be made [6,12,34,37]. Our results provide detailed descriptions of physician actions and attitudes that can be incorporated into the “best evidence model” of decision making [8] to ensure genuine participation of the parents. For example, eliciting and listening to parents’ values, concerns and perspectives of their experience before presenting statistics regarding medical interventions helps to develop the trustworthy parent-physician relationship that facilitates parent participation. Parent-identified strategies; such as pacing one's speech to parents’ needs and supporting parents, sharing the "expert" stance between physician and parent.

Second, the information shared during the antenatal consultation has been shown to help mothers understand what could happen to their baby and thus diminish their concerns [31]. Facilitating parents’ questions and listening to parents’ worries during the antenatal consultations were identified as strengths by parents in our study. Our results confirm that parents want to hear a message of hope [5,19] but still want to face the reality and be told the truth, even though devastating [13]. But more importantly, rather than receiving neutral information as described by Payot et al. [6], our parents described that they appreciated “balanced” information that describes both risks and benefits of treatment options.

Third, as indicated by Kavanaugh, receiving information is not necessarily an indication that the parent will feel involved in the decision-making process [13]. Our study specified that more than just receiving information, parents mentioned the importance of being offered choices of treatment options as facilitating their feeling of being involved in the decision-making process. For example, being told by the physician that resuscitation is the only option can generate a sense of loss of control over the situation and also a feeling of guilt and of being judged for wanting to consider other treatment options such as palliative care.

Finally, our results describe the importance to parents of allowing them time to think about the information received and the treatment options. This is similar to the findings of McHaffie et al. [12] and Kopelman [38]. In our study, offering a follow-up antenatal consultation provided a satisfactory sense of having time to think.

### Strengths and limitations

The qualitative multiple-case study design was chosen to gain the much needed rich description [39] of the parents’ perceptions of the interactions between them and their physician during the clinical antenatal consultation. Granted that case studies do not provide statistically generalizable results, that thematic saturation may not be conclusive based on 5 systems, and results may not represent all physician actions that facilitate parent participation in decision-making. However the rigor of the data collection and analysis as well as the usefulness of this practical first blueprint for physician action in similar contexts constitute a valuable addition to evidence regarding appropriate shared decision making [40].

Interviewing both parents simultaneously during the PAC interview with the aim to obtain a rich and detailed description of parents’ satisfaction of their participation in the decision-making process, it may have precluded finding gender differences in regards of the determinants of parents’ satisfaction.

Although some of the communication strategies reported here have been found in other studies, they were never examined, to our knowledge, from the perspective of parents as they participate in the decision-making process concerning treatment choices for their infant at risk of being born at the limit of viability. These parental descriptions constitute a major strength of our study. The communication strategies linked to these actions are very practical, specific, and useful to physicians seeking shared decision making in this context and potentially in other similar clinical situations.

### Suggestions for future research

Additional research on the perspective of parents involved in decision making in these same, as well as in other specific, situations such as a disagreement between parents and health care providers regarding the best interest of the infant at risk of being born at the limit of viability, would give clinicians still more details on how to facilitate parents’ desired participation.

## Conclusion

The satisfaction of each parent regarding his or her participation in the decision-making process during the clinical antenatal consultation requires the *opportunity* to participate as they desire. The neonatologists can facilitate this opportunity by a multiple of actions throughout the interaction with the parents such as building trust, providing "balanced" information, offering choices, and allowing time to think.

### Clinical implications

Professional societies, including the Canadian Paediatric Society and the American Academy of Pediatrics, strongly support shared decision making in the determination of treatment in the best interests of infants at risk of being born at the limit of viability [4,41]. Their guidelines state that professionals have the responsibility to invite parents to participate and, minimally, to *ask* how the parents want to be involved [41]. However, rather than “ask,” the results of the present study suggest that the physician should ***act*** with parents in ways that facilitate their desired participation.
